# Research on Integrated Innovation Design Education for Cultivating the Innovative and Entrepreneurial Ability of Industrial Design Professionals

**DOI:** 10.3389/fpsyg.2021.693216

**Published:** 2021-08-17

**Authors:** Weifeng Hu, Yue Hu, Yilin Lyu, Yan Chen

**Affiliations:** School of Design, Jiangnan University, Wuxi, China

**Keywords:** innovation and entrepreneurship, integrated innovation design, talents cultivate, education, industrial design

## Abstract

In the industrial design industry, innovation and entrepreneurship made by professional and technical talents will become an important trend in the future development of the economy and society. The great changes in the design industry, such as the diversification and complication of design objects, the relatively restricted knowledge structure of design talents, the lack of entrepreneurial experience, and other reasons, have led to the low success rate of industrial design talent entrepreneurship. The purpose of this research is to analyze and explore the effects of integrated innovation design teaching modes and methods of cultivating the innovative and entrepreneurial abilities of industrial design professionals to help industrial design professionals to improve their success rate of establishing new companies. This research also aims to improve the innovative and entrepreneurial ability of industrial design professionals in terms of integrated innovation and design teaching reform by integrating the theories and methods of industrial design professional training links such as the objectives, teaching contents, teaching modes and methods, performance evaluation of innovation, and design education, combining these with the requirements of the innovative and entrepreneurial ability training of industrial design professionals. In this study, the educational reform of integrated innovation design in a specific Chinese university is taken as an example to carry out theoretical research and practical application. The results of this research show that the theory and method of integrated innovation of design education have made significant contributions to the enhancement of the innovative and entrepreneurial ability of industrial design professionals. This research also reveals a feasible direction for constructing an industrial design innovative and entrepreneurial talent training system that adapts to the transformation and upgrading of the design industry. Meanwhile, methods and teaching modes applied in this research can be promoted in relevant or similar majors to make innovation and entrepreneurship education in colleges and universities more professional, stimulate students' entrepreneurial consciousness, and improve students' abilities of innovation and entrepreneurship.

## Introduction

In the design industry, innovation and entrepreneurship of professional technical personnel will become an important trend in the future development of the economy and society. With the rapid development of emerging industries, the industrial structure has gradually changed. The design object expands from the traditional physical products to the combination of hardware and software, service system design, user experience design, etc. These changes have brought both new opportunities and great challenges for the innovation and entrepreneurship of design talents. The public understanding of innovation is deepening, and the Chinese government has made innovation-driven development strategy the core power of national industrial development. Based on this, interdisciplinary integration and integrated innovation have become an important development direction (Andrei and Pei, 2010; Liu and You, 2018; Zhou, 2020).

The industrial design specialty has become an important carrier of integrated innovation because of its interdisciplinary characteristics (Chen and Zhang, 2020). As a method and means of innovation, design thinking has also infiltrated into many fields and produced gratifying results, which further verified the characteristics of integration and innovation of design discipline (Chen, 2021). The transformation of the role of industrial designers from “artists” to resource integrators who can “extensively participate in the whole process of product development” is sufficient to show that the value of industrial design specialty of multidisciplinary intersection and integrated innovation is gradually recognized and valued by people (Cagan and Vogel, 2018; Gao et al., 2020). Therefore, for the design industry reform or the training of innovative and entrepreneurial ability of industrial design talents, interdisciplinary and cross-boundary integrated innovation is increasingly vital and will become an important development trend in the future (Xie, 2018). In the field of academics, the previous research results fail to provide a systematic theoretical framework for the concepts, processes, elements, and characteristics of the integrated innovation design. It is necessary to make the theory and methods of the integrated innovation design clear and organized systematically. In the field of design education, the Chinese domestic design colleges and universities have realized the tendency of design reform and have been exploring design education reform in recent years.

Through the investigation of Chinese industrial design colleges and universities, it is found that there are still some deficiencies in the talent training mode and curriculum system of China's traditional industrial design education, especially for the improvement of students' innovation and entrepreneurship ability. The specific problems include the following. (1) The training objectives are mainly for the improvement of students' product design ability, lack of requirements for the cultivation of interdisciplinary integration and innovation ability, and pay little attention to the improvement of students' ability of “finding, analyzing and solving problems” and team cooperation ability. (2) The ability training of “product definition” in the early stage of innovative design is not clear enough. (3) In the aspect of the curriculum system, the content of cultivating students' entrepreneurial ability is lacking. (4) In terms of teaching mode, the core design course of the traditional industrial design curriculum system is often taught by a teacher with a professional background in industrial design. This makes it limited to the teacher's professional knowledge background, which is not conducive to the improvement of students' interdisciplinary integration innovation and entrepreneurial ability. (5) In terms of students' course performance assessment, the content of a course assignment report mainly focuses on product design, such as product appearance design, ergonomics analysis, interaction design, etc., which is relative and not comprehensive and systematic.

Therefore, this paper will systematically construct the theoretical framework and methodological system of integrated innovation design from the aspects of the concept, process method, and characteristics of integrated innovation design. The study of the theory and method of the teaching objectives, teaching contents, teaching modes and methods, performance assessments, and other professional industrial design training links of integrated innovation design education, combined with the professional and innovative industrial design talent and entrepreneurial ability training requirements, will finally enhance the innovative and entrepreneurial ability of industrial design professionals in the reform of integrated innovation design education.

## What is Integrated Innovation Design

“Innovation” and “integration” are two key elements of integrated innovation design. Innovation is the main driving force of economic development. For enterprises, innovation means utilizing various new ways to develop resources to meet new market demands. It is classified into product innovation, process innovation, market innovation, supply chain management innovation, and organizational innovation (Schumpeter, 1997). “Integration” is to connect the scattered elements with each other in a certain way so as to realize the resource sharing and collaborative work of the information system, and finally form a valuable and efficient whole (Smailagic and Siewiorek, 1999). Concrete in the field of product or service design and development, integrated innovation design emphasizes rigorous scientific design and development processes and logical steps, adopts a flexible multi-disciplinary design and research method, and always adheres to the “user-centric” principle, the integration of related disciplines, and fielding more resources based on design, engineering, business, or related discipline team building product and service innovation development systems so as to develop strong innovation and conform to the market and social value of a product or service and promote industrial upgrading and innovation ability (Bremner and Rogers, 2013; Cagan and Vogel, 2018).

## Integrated Innovation Education for Industrial Design Professional's Innovative and Entrepreneurial Ability Training

### Objectives and Orientation of Training

Compared with the traditional design education mode of industrial design talent cultivation, integrated innovation design education emphasizes interdisciplinary, integration, and team cooperation. In this research, the knowledge and ability elements necessary for industrial design professionals to establish new companies are obtained by way of surveys, expert interviews, and other methods, and they are integrated into the training objectives of integrated innovation design education. When it comes to cultivating design ability, based on the thinking of integrated innovation, innovative and entrepreneurial ability training is regarded as an important indicator. The training goal of integrated innovation design education is to “systematically conduct user needs and market of target user groups research, define products or services on this basis, integrate multi-disciplinary innovative design methods for innovative design of products or services, provide solutions, and have good team work and communication skills.” It should also “systematically conduct user needs and market of target user groups research [and] define products or services on this basis” to cultivate students' ability to design goal and positioning of the control and enable students to “discover design problems” through trend analysis, user research, and market analysis in the complex design environment and massive data, so as to clarify design objectives and requirements and define products. The “Integrat[ion] [of] multi-disciplinary innovative design methods for innovative design of products or services, provide solutions” emphasizes the integration of multidisciplinary resources and the improvement of problem-solving ability in talent cultivation and enables students to explain the local and overall properties, feasibility, and rationality of the solution from a holistic perspective. The design of products or services requires the cooperation of teams of engineering technology, business, design, and other related disciplines. Designers, due to their professional characteristics, often act as the integrator of the team, so it is very important to cultivate the multidisciplinary integration ability of industrial design talents. “Good teamwork and communication skills” focuses on cultivating industrial design talents with good professional habits, coordination, and communication skills, especially the ability to illustrate the value of abstract design. During the teaching process of integrated innovation design education, students are often organized into groups and carried out in combination with engineering and business teams. In this process, students' communication, expression, and teamwork ability are undoubtedly fully exercised and greatly improved, thus promoting their innovation and entrepreneurship ability (Wei, 2016; Li and Zhang, 2018).

### Teaching Contents Design

**Figure 1:**
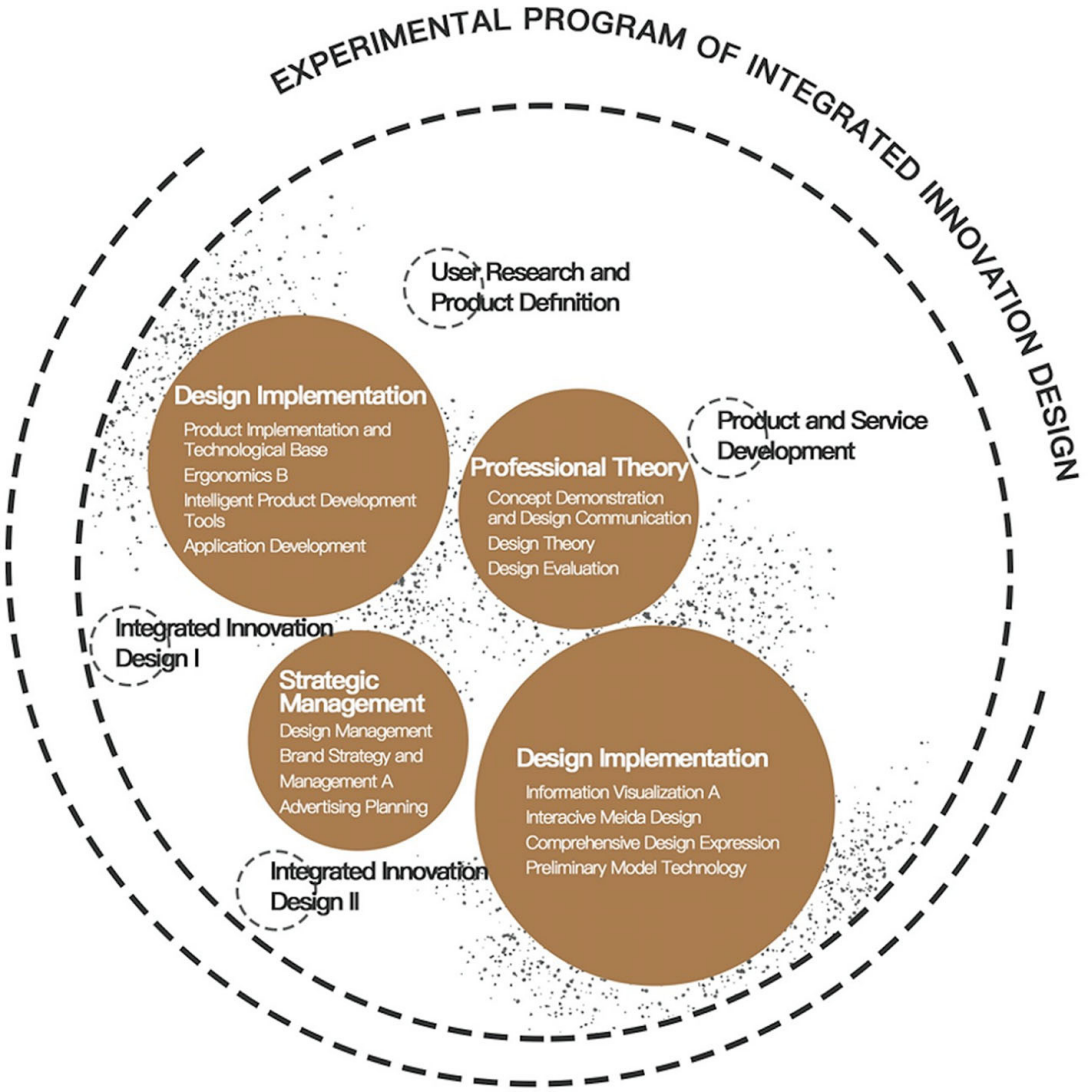
The curriculum of integrated innovation design.

In the process of design teaching contents, according to the training objectives and orientation determined in the early stage, the relevant teaching contents are designed, and they focus on increasingly improving entrepreneurial ability training and striving to cultivate the entrepreneurial consciousness and innovative thinking of industrial design talents (Liao and Bao, 2016). When determining the teaching content, we put emphasis on the knowledge that industrial design professionals are required to command in order to establish new companies, such as market research, user research, intellectual property protection, brand design, teamwork, and other entrepreneurial knowledge. The teaching content is designed from the aspects of professional basic courses, professional core courses, professional elective courses, and intensive practice, as shown in the Figure 1 [Figure 1 is from the “Undergraduate Curriculum Manual 2018–2022” (Unpublished) provided by the Educational Administration Office of School of design, Jiangnan University]. Taking Jiangnan University of China as an example, this paper makes a comparative study on the traditional industrial design curriculum system and the integrated innovation curriculum system by using the comparative analysis method and analyzes the differences, advantages, and disadvantages of the two in the cultivation of innovation and entrepreneurship ability of design talents.

#### Professional Basic Courses

In the professional basic course module, in addition to the cultivation of the basic design ability, the students' consciousness of innovation and entrepreneurship should be enlightened. The professional courses, such as “Design Sketch,” “Color,” “Basic Modeling,” “Design Introduction,” and “Visual Design Foundation” are offered for the training of basic design ability. Compared with the traditional industrial design curriculum system, it is found that the integrated innovation design curriculum system adds the “Monographic Study” course, which is more convenient when arranging the teaching content when flexibly combined with the current industrial changes so as to improve the students' industrial changes adaptability. The integrated innovation design curriculum system reduces the courses of “Mechanical and Electrical basis,” “Industrial Design Expression,” “Computer-aided Industrial Design,” and “Mechanical Drawing and CAD,” and these will be supplemented in professional core courses and elective courses.

#### Professional Core Courses

In the integrated innovation design curriculum system, the professional core courses focus on cultivating students' ability to “found the problem, analyze and solve problems” and improve the students' ability of design innovation and entrepreneurial ability. Four professional core courses are provided, including *User Research and Product Definition, Product and Service Development, Integrated Innovation Design I*, and *Integrated Innovation Design II*. Due to the limitation of professional knowledge, design talents often face problems such as insufficient systematic and comprehensive research on target users and unclear positioning of products or services to be developed, resulting in a low success rate of entrepreneurship. The *User Research and Product Definition* course is designed to broaden students' vision from the traditional restricted design research to user research, brand awareness, and the social economic field, instructing them to comprehensively understand the existing products or services and thus enabling them to acquire the skills of the user research, market research, and product definition. Through the study and application of various user and market research methods of “fuzzy pre design,” we can cultivate the students' ability to explore new products or services, and learn to accurately define new product or service opportunities and formulate design assignments. Meanwhile, through the study, we can help students understand the essence of design basis, get familiar with the team decision-making mode, and cultivate in them the ability to establish scientific decision-making ability, design thinking modes, and good design habits so as to enhance students' ability of innovation and entrepreneurship. Through this course, students should understand how to comprehensively recognize the attributes of products or services and learn to interpret their rationality and adaptability in the social, economic, and cultural environment. They should also understand what the “fuzzy product pre stage” is and master a variety of user and market research methods of the “fuzzy pre stage,” including case analysis, desktop research, questionnaire survey, user interview, field survey, understand what the design task book is and its application approach and value. At the same time, on the basis of preliminary familiarity with team decision-making mode, students should understand and master the new product or service definition method centered on user demand in the early stage of product development, and finally develop a complete design task book. Systematic learning of user research and market research, as well as the ability to find product opportunities and define products on this basis, is one of the key components of innovation and entrepreneurship. The systematic learning of *User Research and Product Definition* can effectively improve students' innovative and entrepreneurial ability.

Traditional industrial design talents still remain in the design of products design, while the design industry environment has undergone significant changes. And the scene of innovation and entrepreneurship is increasingly complex. Therefore, the cultivation of students' awareness of problems and systematically learning the design and development theories and methods of products and services will improve students' ability to adapt to the complex design industry environment and their ability to innovate and start a business. *Product and Service Development* is a professional core course whose purpose is to learn about problem-oriented integrated innovation design methods. It is a course that centers on practical skills and theoretical knowledge. Through this course, students can improve their product design innovation ability and then enhance their possibility of entrepreneurial success. Through the targeted team project practice, the course enables students to understand the basic principles and methods of developing products or services, training students to rationally choose and use the methods of early conceptual design and late conceptual demonstration based on user research and according to the requirements of the product design demands. This course is also to train students' oral and written expression ability while cultivating professional skills of solving practical problems so as to obtain the ability to complete the implementation of design tasks. Through the study of this course, students should have the ability to implement the design assignment based on user research or according to the requirements of the product design specification; understand and acquire the methods and tools of designing different tangible and immaterial products and the corresponding implementation, including the production of a low-fidelity functional model, VOA (Value Opportunity Analysis), weighted matrix, stakeholder analysis, etc. They should also understand and rationally choose different product and service assessment methods according to the specialized design demands. In addition, through this course, students should learn how to use research and design expression as two mutually reinforcing tools for communications and arguments, get more familiar with and accustomed to the team decision-making mode, and then enhance the ability of industrial design talent entrepreneurship.

**Figure 2:**
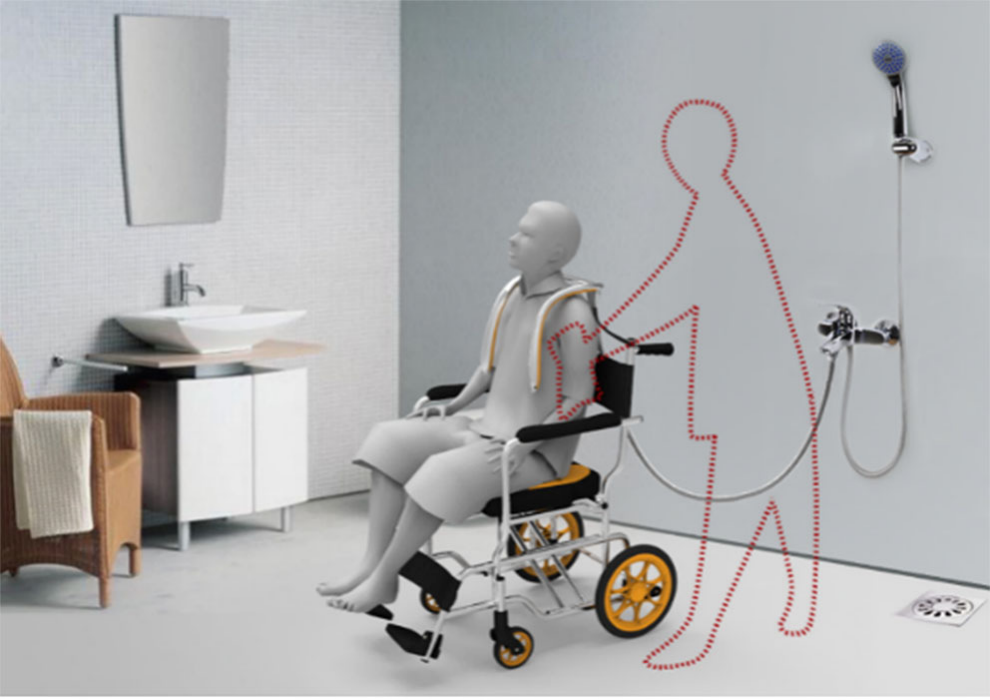
Integrated innovation coursework: bathing and defecating chair for the elderly.

Integrated innovation ability is becoming a key ability factor to assess the success of innovation and entrepreneurship of design talents. It is an effective way to improve the success rate of entrepreneurship of design talents by systematically cultivating students' ability to integrate design, engineering, business, and other related disciplines. *Integrated Innovation Design I* is a professional core course that helps the student to grasp and skillfully use user research and product definition, such as the foundation design method, according to the requirements of the different projects to guide students to use cultural innovation, sustainable development, interaction design, and different design methods through the problem-oriented virtual subject or the actual project. Through the settings of different themes, students can understand the social, cultural, economic, and other macro factors that influence the judgment of design values and think about different design criteria under different backgrounds, including forming a judgment of the practical significance of the project itself. Through the study of this course, students should have the ability to choose the design methods according to the project nature, and grasp the cultural innovation, sustainable development, or interaction design and other design tools based on different design methods such as ICA (interpret cultural artifacts), the ICB (interpret cultural behavior), 3D, 3R, PSS, information architecture, user interface design, etc. Through learning different methods and tools, students are required to reasonably adopt the design tools according to the demands of the project. Finally, through the study of this course, students should also learn to comprehensively assess and fully demonstrate the design results from the two aspects of user needs and macro-social significance, and write a complete project report. *Integrated Innovation Design I* is helpful to improve students' ability to understand integrated innovation and increase the possibility of successful innovation and entrepreneurship of design talents when they are faced with complex design situations (As shown in Figure 2, the “Bathing and defecating chair for the elderly” is an *Integrated Innovation Design I* coursework, which has won the national invention patent of China).

**Figure 3:**
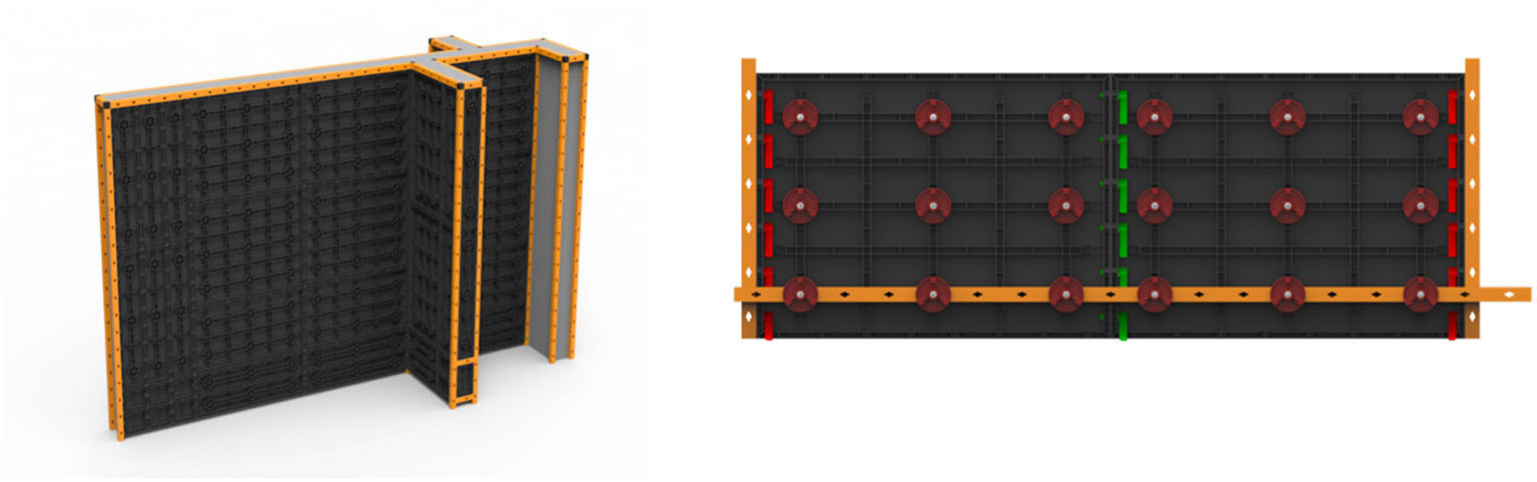
Design of recyclable construction formwork-*integrated innovation design* coursework.

The *Integrated innovation design II* course is a professional core course that aims to make students master different design methods, such as user research, product definition, and product development. On the basis of this course, students can understand and try to use the concepts and methods of system design, and have the comprehensive ability to propose reasonable and feasible product solutions according to user needs, actual social environment, or business conditions or can even demonstrate the corresponding business model they can also demonstrate a preliminary understanding of project management, design management, and other knowledge and skills. This course is intended to complete the method learning and basic ability training, and it is a core course for students to try to change their roles from students to professional designers. In this course students must (1) fully grasp the application of various methods and tools in the whole process of product development from finding problems, analyzing product opportunities, and to solving problems; (2) understand and simply use advanced design methods such as system design and business model; (3) have the ability to propose reasonable and feasible product solutions according to user needs, practical social environment, and business conditions, and even the corresponding business model, and show an overall demonstration of the solution in the form of a written report; and (4) on the basis of continuing the team project, show a preliminary understanding of project management, design management, and other knowledge and skills. This course will not only further improve the students' ability of integration and innovation but also enhance their ability to build a business model and improve project management, thus increasing the success possibility of design talents' innovation and entrepreneurship (Figure 3 shows the Recyclable Building Formwork Design is an *Integrated Innovation Design II* course work, which has obtained the Chinese national invention patent and has been used to carry out the market promotion application).

In addition to the four core design courses that focus on the cultivation of students' integrated innovation ability and have an obvious logical progressive relationship, the core courses of integrated innovation design also include “Information Visualization Design,” “Product Realization and Technology Foundation,” “Concept Demonstration and Design Communication,” and other courses that focus on the cultivation of students' relevant technology realization and design communication ability; included are also “Design Management,” “Brand Planning and Management,” and other courses to enhance students' ability of brand planning and management. However, the traditional industrial design curriculum system is more inclined to the deepening of some specific design fields, such as “Interaction Design Principles and Methods,” “User Interface Design,” “Interaction Design Practice,” and so on. There are also courses to improve the ability of product design, such as “Material Technology,” “Product System and Service Design,” “Folk art Investigation and Innovative Design,” “Special Research,” etc. Compared with the curriculum system of the integrated innovative design, the curriculum system of the traditional industrial design is relatively weak in the cultivation of students' innovation and entrepreneurship ability.

#### Professional Selective Courses

The professional selective course module is an extension and knowledge expansion part of the professional core courses. Therefore, more selective courses will be set up in the professional selective course module of the integrated innovation design curriculum system. Compared with the traditional industrial design curriculum system, there are more professional elective courses in the integrated innovation design curriculum system (there are 31 elective courses), covering a wide range of knowledge fields, involving commercial market, media, space display, graphic design, photography, art practice, computer program development, aesthetic, design expression, service design, advertising planning, ergonomics, animation, intelligent products, furniture, and other fields, to provide students with more choices and expand the knowledge fields that students need for innovation and entrepreneurship. There are relatively few traditional industrial design professional elective courses (there are only 17 professional elective courses), and the curriculum is more specific to the design field, such as “Product Sketch,” “Preliminary Model Technology,” “Comprehensive Design Expression,” and so on, There are also expanding courses in other design fields, such as “Two-dimensional Animation Design,” “Information Design,” “Ancient Utensil Design Research,” and so on, and knowledge expansion courses for interaction design, such as “Software Engineering,” “Interaction Design Technology,” etc. For specific design method courses, there are courses like “Design Psychology,” “Design Demonstration,” “Product Semantic Design,” “Kansei Engineering,” and so on. From the setting of these elective courses, it can be found that the purpose of traditional industrial design elective courses is to strengthen the students' design ability training and design knowledge learning, while the courses for students' innovation and entrepreneurship ability training are still relatively lacking.

#### Intensive Practical Training

The intensive practical sessions focus on the comprehensive application of knowledge and methods learned in the early stage, so the cross-design and practical sessions are set up in the integrated innovation design curriculum system, as well as a practice inspection part. The cross-design and practice courses focus on cross-disciplinary and team cooperation, so when students organize teams and choose instructors, they should try to choose multidisciplinary teams based on the topic itself. In addition, it also provides students with opportunities to communicate and learn with successful designers so as to enhance students' innovation and entrepreneurship ability.

Based on the analysis of the above-integrated innovation design curriculum system, and comparing the traditional industrial design curriculum system with the integrated innovation design curriculum system, we can find the following conclusions:

1. The traditional industrial design curriculum system pays more attention to the cultivation of students' product design ability. The integrated innovation design curriculum system is based on the consideration of the students' design ability training and pays more attention to the cultivation of the students' integrated innovation and entrepreneurship ability.
2. The traditional industrial design curriculum system emphasizes the knowledge development and deepening in a specific design field in the course setting. The integrated innovation design curriculum system has a more obvious logical progressive relationship, following the path of finding, analyzing, and solving problems.
3. Compared with the integrated innovation design, the traditional industrial design professional elective course is less, the knowledge coverage of the course is narrow, and the course is inclined to study the theory, method, and tools of the specific product design field. The integrated innovation design professional elective course is more, and the knowledge field is also relatively wide, which provides more choices for students and expands the knowledge fields required by students' innovation and entrepreneurship.

Therefore, combining with the above analysis, we can find that the integrated innovation design curriculum system has a significant advantage in the cultivation of innovation and entrepreneurship ability of industrial design talents.

### Teaching Methods and Models

#### Double Teacher System Teaching and Team Cooperation of Students

Integrated innovation design involves a wide range of knowledge, which has higher requirements for the knowledge field and design practice ability of teachers. Therefore, the professional core courses are jointly taught by teachers with rich design practice experience and corporate mentors with rich entrepreneurial knowledge so as to maximize teaching effectiveness and improve students' innovation and entrepreneurial ability. The design professors design the teaching content and set the schedule of the course as a whole and give targeted knowledge lectures at key course nodes; the corporate mentors provide lectures and tutorials on entrepreneurship knowledge with their own entrepreneurial experience. Together, the two teachers combine their rich design practice experience and entrepreneurial experience to jointly review and guide the course assignments. In addition, in some aspects, it is also necessary to invite other industry experts to provide targeted knowledge lectures and guidance. For example, in the late stage of the *Product and Service Development* course, it is necessary to apply for the patent of the design and development scheme, and then invite patent application engineers from patent offices to give a targeted lecture on the relevant requirements and data preparation of the patent application. In order to raise the students' awareness of intellectual property protection and their ability to protect intellectual property, a targeted lecture is necessary. Since the core curriculum of integrated innovation design is often based on a real design project, it is essential for students to form teams to simulate the real corporate project development team. Teams are also a useful means to practice teamwork and project communication skills. The double teacher system teaching mode and the team cooperation method will greatly enhance students' teamwork skills and improve their design innovative and entrepreneurial abilities.

#### Teaching Model Based on Design Practice Project

The professional core courses of integrated innovation design usually aim at real enterprise design projects. First of all, the selection of design practice projects should be fully combined with the training objectives and schedule of the course. And the appropriate design projects should be integrated into classroom teaching. Appropriate projects often have the following characteristics. First, item difficulty is moderate. The choice of design objects should be in accordance with courses. For example, students can choose some projects with a relatively low difficulty of design objects in the courses of *User Research and Product Definition* and *Product and Service Development*, such as lamps, lanterns, and furniture. In courses like *Integrated Innovation Design I* and *Integrated Innovation Design II*, students can choose more difficult and relatively more complex products, such as medical equipment, transportation, etc. Secondly, if it is an actual project of an enterprise, it is necessary to communicate with the enterprise in advance and coordinate with the schedule of the course in terms of the project development cycle and the form of project submission. Thirdly, enterprises would send their engineers and technicians to participate in the assessment of students' design proposals and provide technical supports on a regular basis. In addition, the engineering and commercial teams of enterprises are required to supplement the faculty of universities as much as possible. We strive to have designers and engineers from enterprises participate in students' design reviews and project proposal discussions regularly. The teaching mode based on real “design practice projects” will greatly enhance the communication and cooperation between students and engineers and business marketers, which will greatly increase the probability of students' success in innovation and entrepreneurship (Zhang and Guo, 2016).

#### Teaching Mode Based on the Key Time Node Lecture

Traditional industrial design courses are mostly carried out in the form of combining design theory and design practice, and the form of “theory in the early stage + design practice in the later stage” is often adopted in class. This combination will often bring about a disconnection between theory and practice. When students do the post-design practice, they have no deep impressions and forget most of the theoretical knowledge in the early stage. The professional core courses of integrated innovation design education in this paper are taught in the form of “lectures at key points in time” in which the lectures with targeted theoretical knowledge are integrated into the design practice process at the corresponding points in time, thus greatly enhancing the effect of combining design theory and practice and avoiding the boring and lengthy theoretical lectures in the early-stage process. For example, in the teaching process of the *User Research and Product Definition* course, “design research methods” will be explained in the stage of the practical project when design research is needed so that students can immediately apply the theories and methods learned from the lectures in design research, thus improving the design theory. In this way, students can immediately apply the theories and methods learned in the lectures to design research, thus improving the combination of design theory and practice and enhancing students' innovative and entrepreneurial ability.

### Principles and Criteria for Evaluating the Performance of Teaching Programs

As the professional core courses of integrated innovation design education mainly use students to form a team to complete the project, how to set up the learning assessment mechanism is related to whether it can motivate the team and individual learning enthusiasm. At the same time, course performance is also an important indicator to evaluate students' innovative and entrepreneurial ability.

The professional core course of integrated innovation design is generally evaluated by two teachers, and there are usually 30 students in each class. The specific calculation formula of each student's score is as follows:

$$\begin{array}{l} \text{y}=\text{x}1\times 70\text{\%}+\text{x}2\times 20\text{\%}+\text{x}3\times \;10\text{\%}. \end{array}$$

Here, y represents the total score of the course, x1 represents the homework score, x2 represents the classroom performance score, and x3 represents the attendance score.

Course grades are mainly composed of student homework report grades and the classroom performance score and attendance grades, with their proportional relationship adjusted according to different course characteristics, often with the homework report grades occupying 70% of the total score of the course.

The specific types of students' homework reports are as follows. First, there is the part that reflects students' ability to find problems, analyze problems, and define products in the fuzzy early stage of innovative design. The submitted reports include user research and market research reports, product assignment, etc. Second, there is the part that reflects the students' interdisciplinary product innovation design ability. In the part of hardware product design, students are required to submit product design rendering, product disassembly drawing, product technical principle, and material structure analysis report, product ergonomics analysis, product use scenario analysis, etc. In the part of software product design, students are required to submit the function requirement list, information architecture, user interface design, etc. Third, there is the part that reflects students' innovative design ability of service system based on integrated thinking. Students are required to submit service system analysis, service design touchpoint analysis, stakeholder analysis, and other content. Fourth, there is the part that asks for reflection on the ability of students to create a new company. Students are required to submit product business model analysis, intellectual property application documents, company brand image design, and publicity packaging material design. Last, there is the part reflecting the students' teamwork ability. Students are required to submit a description of the tasks assigned by the team members when completing the course assignment.

The results of the homework report will take the difficulty of homework selected by each group of students into consideration. If the same course is composed of multiple different homework scores, it will also be weighted according to the difficulty, complexity of homework and the importance of the corresponding ability investigated, so as to get the final homework report results. Collaboration among group members is also important for the project, but because group members contribute differently to the project and attendance varies, the grades are not always the same for all members of the same group, and differentiated grades motivate the best group members and spur those who do not contribute as much, and motivate group members to participate in the project. The assessments of performance are usually discussed by the design professional teachers and enterprise tutors who undertake the task of class according to the homework report, attendance, and daily performance of each group so as to minimize the influence of teachers' personal preferences on the performances of the students.

Specific assignment and report grading criteria are as follows:

1. Level A: The process is perfect and reasonable. The application of tools and methods is correct. The design result is innovative, feasible, and typical in terms of market potential. The team has excellent team reporting and communication skills. The report follows reasonable logic and has clear chapters and smooth sentences.
2. Level B: The process is reasonable. The application of tools and methods is relatively correct. The design result is innovative, feasible, and typical in terms of market potential. The team shows good team reporting and communication skills. The report follows reasonable logic and has clear chapters and smooth sentences.
3. Level C: The process is basically reasonable, and the application of tools and methods is basically correct. The innovation, feasibility, typicality, and market potential of the design result are moderate. The team shows good team reporting and communication skills. The logic of the report is basically reasonable, and the chapters and sentences are acceptable.
4. Level D: The process is unreasonable. The application of tools and methods is improper. The innovation, feasibility, typicality, and market potential of the design results are at a relatively low level. The team shows ordinary team reporting and communication skills. There are problems in the report logic, chapters, and sentences.
5. Level E: The process is seriously unreasonable, and serious problems exist in the application of tools and methods; The innovation, feasibility, typicality, and market potential of the design results are at a low level. The team shows poor team reporting and communication skills. There are serious problems in the report logic, chapters, and sentences.

The classroom performance is mainly evaluated by the teacher in combination with the students' classroom report, such as the language expression ability of the report, the enthusiasm of teamwork, the ability to solve problems, the ability of innovation and entrepreneurship, etc. due to the discipline characteristics of design major, the performance evaluation of this part is relatively subjective.

In terms of attendance results, generally, 10 instances of attendance will be carried out randomly in the classroom, and the attendance results will be calculated according to the attendance times of students.

The establishment of scientific and reasonable teaching curriculum performance assessment standards can greatly stimulate students' learning enthusiasm and systematically investigate the effect of students' innovative and entrepreneurial ability training.

### The Practice and Effectiveness Verification of Integrated Innovation Design Education

Since the implementation of the integrated innovation design education in Jiangnan University in 2012, the practice has proved that the education theory and method effectively improve the innovation and entrepreneurship ability of undergraduates majoring in industrial design and product design. Since 2015, the integrated innovation design education, which was just a pilot in the early stage, has been gradually promoted in other majors (especially industrial design and product design majors) in the design school of Jiangnan University, such as the promotion of the core design courses like “User Research and Product Definition” and “Integrated Innovation Design” in other majors. By using the methods of statistical analysis and comparative research, according to the relevant data of the design school of Jiangnan University in recent 5 years, this paper concludes that the improvement of the innovation and entrepreneurship ability of students in the industrial design department, product design department and integrated innovation design experimental class of Jiangnan University can be reflected in three aspects.

**Figure 4:**
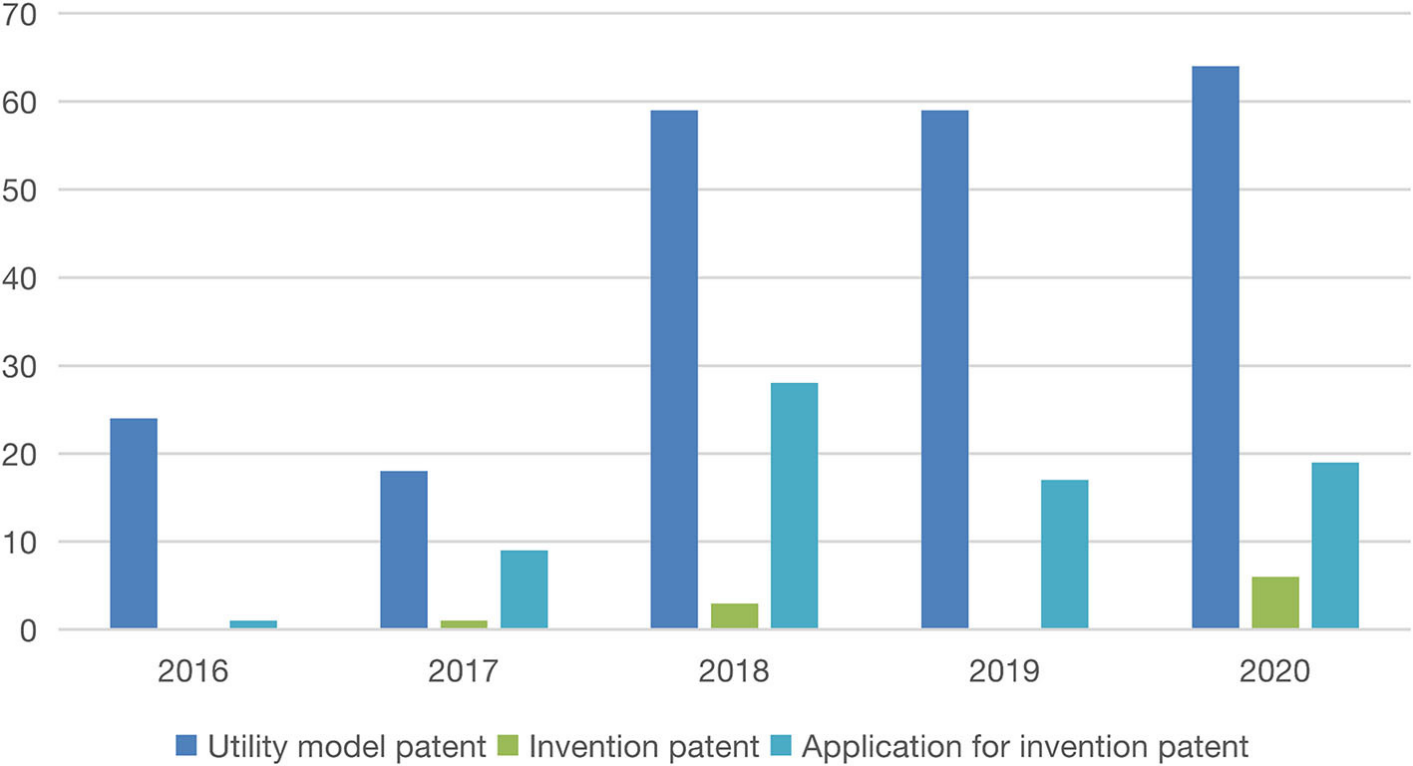
Statistical chart of patent application and authorization.

First, the number of patent applications and authorizations are important reference values to reflect the innovation ability of teachers and students. According to the data provided by the scientific research office of the design school of Jiangnan University, the number and quality of patents applications and authorizations obtained by teachers and students in the industrial design department, product design department, and integrated innovation experimental class have been on a rising trend in the past 5 years (as shown in Figure 4). In 2016, they obtained 24 utility model patents, 0 invention patents, and 1 invention patent application. In 2017, they obtained 18 utility model patents, 1 invention patent, and 9 invention patent applications. In 2018, there were 59 utility model patents, 3 invention patents were authorized, and 28 invention patents were applied. In 2019, there were 59 utility model patents, 0 invention patents were authorized, and 17 invention applications were applied. In 2020, there were 64 utility model patents, 6 invention patents were authorized, and 19 invention patents and 2 international invention patents were applied. It can be seen that in the past 5 years, the number of patents has been increasing, especially the number of invention patent authorization and applications reflecting innovation ability has increased rapidly, and there was an international invention patent application in 2020. This proves to a certain extent that the innovation ability of students and teachers of the industrial design department, product design department, and integrated innovation design experimental class in Jiangnan University has improved.

Second, the graduates of the industrial design department, product design department, and the integrated innovation design experimental class are very popular with enterprises. Many students got jobs in Tencent, Haier, and other large enterprises to engage in product design, interactive design, and other innovative design work. In addition, they also won the IDEA international design award in the United States, the iF design and Red Dot design awards in Germany, the Guangbao design award in Taiwan, the Asian design year award, and other international high-level design awards. The students of the industrial design department, product design department, and the integrated innovation design experimental class actively participate in the innovation and entrepreneurship project of Jiangnan University, which also reflects that the students have high enthusiasm for innovation and entrepreneurship. For example, in 2019, the teachers of the industrial design department, product design department, and integrated innovation design experimental class led students to obtain 14 items of Jiangnan University Students' Innovation Training Program projects. A total of 56 students participated in these projects, including four national projects and two provincial projects. A total of 22 academic papers or research reports were published, and three utility model patents and one appearance patent were granted. In 2020, they got seven projects of the Jiangnan University student innovation training program. A total of 28 students participated in the projects. Of these, five were awarded provincial key projects. The main reason for the reduction in the number of projects in 2020 was the new Coronavirus epidemic. These projects are currently in progress. In 2021, they got 15 innovation and entrepreneurship projects from Jiangnan University. A total of 60 students participated in these projects, and these projects are also in progress.

Third, among these students, there are also some successful cases of outstanding performance in innovation and entrepreneurship. For example, the children's hand sanitizer equipment developed by Wang Huandong from the experimental class of integrated innovation design in Jiangnan University in the course of “Product and Service Development” shows strong innovation. The children's hand sanitizer equipment was adopted by China Shandong Liangfu Pharmaceutical Co., Ltd., paid 50,000 Yuan for its intellectual property rights, and reached a cooperation agreement. The research on Tesla electric vehicle in the course of “User Research and Product Definition” by the students of the integrated innovation design experimental class of Jiangnan University has been highly praised by Tesla, and their homework video on Tesla attribution analysis has been uploaded to the official website of Tesla.

## Discussion

### Advantages and Disadvantages of Innovation and Entrepreneurship of Industrial Design Talents

The industrial design profession grows with the development of the industrial economy, which also determines that its professional development direction is closely related to the development of the industrial economy (Cao et al., 2019). The design objects of industrial design majors, from the initial product appearance design to the product functional structure design and to the current intelligent product design, the cultivation of talents always follows the development direction of the industrial economy. Industrial design talents have received systematic design thinking and design method training and have certain design innovation abilities. Therefore, industrial design professionals have certain advantages of innovation and entrepreneurship. However, there are also some disadvantages (Guo, 2018). These disadvantages cause the problems of the low success rate of innovation and entrepreneurship of industrial design talents. For example, industrial design talents do not have a deep enough understanding of knowledge and research methods related to users, markets, and business, resulting in the inability to truly access the core pain points of users and the lack of in-depth understanding of market trends, thus failing to discover the direction of product innovation with commercial value (Xia, 2019). Industrial design talents do not have deep enough knowledge about engineering technology, resulting in innovative design concepts not being better manufactured by mass production, and they are inexperienced in enterprise management and intellectual property protection so as to become a barrier for them to carry out successful innovation and entrepreneurship. Based on the above analysis, industrial design talents, as an important component force of China's innovation and entrepreneurship, have received strong support from the government and industry, which is also an important trend for the future development of the economy and society. How to enhance the integration and innovation ability of industrial design talents so that industrial design talents have the ability to integrate the knowledge of engineering technology, business, management, and other disciplines to conduct innovation and entrepreneurship is an important research direction, and it is also an important goal of industrial design talents training (Yuan, 2018; Sun, 2020).

### The Necessity of Innovation and Entrepreneurship Research Based on Integrated Innovation Design

Integrated innovation design, compared with the traditional industrial design, places emphasis on discipline overlapping and integration ability, emphasis on design thinking as the main line, integration of related subjects such as engineering, business, market to build a product and service innovation development system. The integrated innovation design education is a very effective education model for cultivating the innovative and entrepreneurial ability of design talents. The current research on integrated innovation design at home and abroad has two main research results. First, an integrated innovation design about product and design development, such as for automotive design (Xiao, 2017; Xiao et al., 2020), leather products (Sun, 2020), emphasizes the integration of design, the engineering and business discipline teams based on the overall process of product development for overall product innovation and development with the aim of achieving a more optimized product design and development process and higher quality design output. Second, research and practice on teaching methods and teaching models of design disciplines based on integrated innovation design, such as visual communication (Wang and Zhang, 2017), product design (Zhu, 2016; Qiu, 2018; Chen and Yu, 2020), etc., and research on integrated innovation design education have been conducted for the integrated innovation education model and methodological system of undergraduate students in these majors. Existing research on integrating innovation design education in the development of innovation and entrepreneurship of industrial design talents is still insufficient. Therefore, this research carries out the corresponding research according to the innovative and entrepreneurial ability demands of industrial design talents to make up for the lack of existing research.

Integrated innovation design has advantages over traditional industrial design education models for the cultivation of design talents' innovation and entrepreneurship. Integrated innovation design education emphasizes interdisciplinary and multidisciplinary integration and teamwork, and it provides systematic learning and practical training in user research, market research, and product definition in the early stage of design. In addition, it provides a business model design, intellectual property protection, and design management in the late stage of product design development, which can systematically enhance the innovative and entrepreneurial ability of design talents.

## Conclusion

This paper defines the concept of integrated innovation design, aiming to improve the innovative and entrepreneurial ability of design talents from the perspective of education. This study aims to train industrial design talents' ability to bring ideas to the market. It studies and gives specific solutions to teaching objectives, teaching contents, teaching methods and modes, curriculum performance assessment, making theoretical and practical contributions to the cultivation of innovative and entrepreneurial ability of design talents. The innovation ability of industrial design talents is reflected in the ability to create, define, discover, and leverage opportunities. Its procedures include idea conception and successful product development with the goal of enabling the company to bring new products to market. The development of products, services, and solutions forms the core of a company's competitive strategy. In terms of the training target setting, according to the knowledge and ability elements necessary for the establishment of a new company by the industrial design professionals, the goal and orientation combining the design ability of the students with the cultivation of innovative and entrepreneurial ability are set.

In terms of teaching content, the knowledge needed by the talents of industrial design, especially to create a new company, correspond with practical opportunities required by the enterprises, such as market research, user research, intellectual property protection, brand design, and teamwork, are set systematically. In terms of teaching modes and methods, the double teacher teaching mode is adopted, and the effective integration of professional theoretical learning and entrepreneurial practice is emphasized. The team cooperation, research, and design practice based on the real enterprise design project is also given priority, which improves the ability of team cooperation and project communication needed by the design talents to start their business. The principles and standards of performance assessment are set scientifically and reasonably, which inspire the students to learn and scientifically assess the effectiveness of the cultivation of students' innovative and entrepreneurial ability.

The feasibility of future research is as follows. On the one hand, this study mainly analyzes the innovative and entrepreneurial ability training of industrial design talents based on integrated innovation design. For other relevant majors, such as digital media, visual communication, and environmental art design, further research is needed. On the other hand, this study aims at the research on the cultivation of innovative and entrepreneurial ability of industrial design talents through integrated innovation and design education. It is necessary to track the observation and assessment of long-term innovation and entrepreneurship effect of industrial design majors who have received this training system and adjust and improve the long-term training mode according to the assessment results. That is also the direction of our continuous efforts in the future.

## Data Availability Statement

The original contributions presented in the study are included in the article/supplementary material, further inquiries can be directed to the corresponding author.

## Ethics Statement

Ethical review and approval were not required for the study on human participants in accordance with the local legislation and institutional requirements. The patients/participants provided their written informed consent to participate in this study. Written informed consent was obtained from the individual(s) for the publication of any potentially identifiable images or data included in this article.

## Author Contributions

YH, YL, and YC: material preparation, data collection, and analysis were performed. The first draft of the manuscript was written by WH and all authors commented on previous versions of the manuscript. All authors contributed to the study's conception and design, and read and approved the final manuscript.

## Conflict of Interest

The authors declare that the research was conducted in the absence of any commercial or financial relationships that could be construed as a potential conflict of interest.

## Publisher's Note

All claims expressed in this article are solely those of the authors and do not necessarily represent those of their affiliated organizations, or those of the publisher, the editors and the reviewers. Any product that may be evaluated in this article, or claim that may be made by its manufacturer, is not guaranteed or endorsed by the publisher.
